# Fluoroquinolone Resistance in Penicillin-resistant *Streptococcus pneumoniae* Clones, Spain

**DOI:** 10.3201/eid1010.040382

**Published:** 2004-10

**Authors:** Adela G. de la Campa, Luz Balsalobre, Carmen Ardanuy, Asunción Fenoll, Emilio Pérez-Trallero, Josefina Liñares, the Spanish Pneumococcal Infection Study Network G03/103

**Affiliations:** * Instituto de Salud Carlos III, Majadahonda, Madrid, Spain;; † Hospital de Bellvitge, L'Hospitalet de Llobregat, Barcelona, Spain;; ‡ Hospital Donostia, San Sebastian, Guipúzcoa, Spain

**Keywords:** DNA topoisomerases, fluoroquinolones, pneumococcus, research

## Abstract

Among 2,882 *Streptococcus pneumoniae* sent to the Spanish Reference Laboratory during 2002, 75 (2.6%) were ciprofloxacin-resistant. Resistance was associated with older patients (3.9% in adults and 7.2% in patients >65 years of age), with isolation from noninvasive sites (4.3% vs. 1.0%), and with penicillin and macrolide resistance. Among 14 low-level resistant (MIC 4–8 µg/mL) strains, 1 had a fluoroquinolone efflux phenotype, and 13 showed single ParC changes. The 61 high-level ciprofloxacin-resistant (MIC >16 µg/mL) strains showed either two or three changes at ParC, ParE, and GyrA. Resistance was acquired either by point mutation (70 strains) or by recombination with viridans streptococci (4 strains) at the topoisomerase II genes. Although 36 pulsed-field gel electrophoresis patterns were observed, 5 international multiresistant clones (Spain^23F^-1, Spain^6B^-2, Spain^9V^-3, Spain^14^-5 and Sweden^15A^-25) accounted for 35 (46.7%) of the ciprofloxacin-resistant strains. Continuous surveillance is needed to prevent the dissemination of these clones.

## Abstract 2

Of 75 clones isolated, 1 had ciprofloxacin efflux, and 74 had mutations at the DNA topoisomerase gene.

Resistance of *Streptococcus pneumoniae* to multiple antibacterial agents, including β-lactams, macrolides, tetracyclines, and co-trimoxazole, has emerged worldwide in the 1980s and 1990s (*1**–**3*) and has emphasized the need for new therapeutic alternatives, such as newer fluoroquinolones. Older fluoroquinolones, such as ciprofloxacin and ofloxacin, have been widely used in the last 2 decades, but their activity against gram-positive pathogens is limited. Newer fluoroquinolones, such as levofloxacin, gatifloxacin, moxifloxacin, and gemifloxacin, have enhanced activity against most respiratory pathogens, and some are being more widely used to treat respiratory tract infections. Therefore, the emergence of fluoroquinolone-resistant *S. pneumoniae* strains, although worldwide prevalence is low, is a concern to clinicians who manage respiratory tract infections. A global surveillance study from 1998 to 2000 included 8,882 pneumococci isolated from blood and sputum samples that were collected from centers in 26 countries. The study showed a 1.1% prevalence of ofloxacin-resistant strains, although in some places higher values of nonsusceptible ofloxacin strains were observed: 10.1% in Israel, 14.1% in Japan, and 22.3% in Hong Kong (*1*). Ciprofloxacin-resistant strains isolated from patients with community-acquired respiratory tract infections have been reported in Spain (3.0% [*4*] and 7.1% [*5*]), Canada (1.7% [*6*]), and the United States (1.4% [*7*]).

Fluoroquinolone resistance is higher among related viridans group streptococci (VGS) isolated from blood (*8*). Increased fluoroquinolone use correlates with an increase in the prevalence of ciprofloxacin resistance (*4**,**6*). Prior fluoroquinolone administration is a risk factor for resistant strain selection, as observed for infections caused by *S. pneumoniae* (*9**–**12*) and VGS (*13*). A study from our group showed ciprofloxacin-resistant pneumococci emerging in a patient who had received long-term ciprofloxacin therapy to treat persistent bronchiectasis with *Pseudomonas aeruginosa* infection (*14*).

Bacterial resistance to quinolones occurs mainly by alteration of their intracellular drug targets, the DNA topoisomerase IV (ParC_2_ParE_2_) and DNA gyrase (GyrA_2_GyrB_2_) enzymes. The pneumococcal *parC* and *parE* genes are homologous to *gyrA* and *gyrB*, respectively (*15**,**16*). Genetic and biochemical studies have shown that fluoroquinolones target primarily topoisomerase IV and secondarily DNA gyrase in *S. pneumoniae* (*16**–**19*). Resistance mutations are localized in the quinolone resistance-determining regions (QRDRs) of ParC, ParE, and GyrA. Low-level ciprofloxacin-resistant strains had mutations altering the QRDR of one of the two subunits of topoisomerase IV: S79 or D83 of ParC (*16**,**17**,**20*) or D435 of ParE (*21*). High-level ciprofloxacin-resistant strains had changes affecting both QRDRs of ParC and GyrA (S81, E85) (*16**,**17*) or ParE and GyrA (*21*). Genetic transformation experiments showed that single *parC* mutations confer low-level ciprofloxacin resistance, and that once the cells have acquired this phenotype, transforming to a higher level of resistance was possible by using DNA containing the *gyrA* QRDR from the high-level ciprofloxacin-resistant strains (*16**,**17*). The description of recombinant strains with a mosaic structure in their DNA topoisomerase genes (*22**–**24*) has established that fluoroquinolone resistance in *S. pneumoniae* can be acquired by horizontal gene transfer as well as by point mutation. VGS, which share the same mechanisms of resistance (*13*), are donors in the horizontal transfer to pneumococci (*25*) and act as a reservoir of fluoroquinolone resistance. To investigate the epidemiology of fluoroquinolone-resistant pneumococci, ascertain the possible dissemination of international clones, and determine the incidence of resistant strains originated by interspecific horizontal transfer, we characterized all ciprofloxacin-resistant *S. pneumoniae* strains sent to Spanish Reference Laboratory during 2002.

## Materials and Methods

### Bacterial Strains, Serotyping, and Susceptibility Tests

We studied 2,882 *S. pneumoniae* strains submitted from 78 hospitals nationwide to the Spanish Reference Laboratory during 2002. Of the submitted strains, 1,904 (66.1%) were isolated from adults and 978 (39.1%) from children. The origin of isolates was as follows: 1,453 (50.4%) from blood or other sterile sites, 691 (24.0%) from respiratory tract, 231 (8.0%) from eye swabs, 205 (7.1%) from ear swabs, and 302 (10.5%) from other sites. Strains were confirmed as *S. pneumoniae* by standard methods, and serotypes were determined by a quellung reaction. Susceptibility tests, performed initially by the agar-dilution method, selected 85 strains (one isolate per patient) with ciprofloxacin MIC > 4 µg/mL. The susceptibility of these 85 strains was repeated twice by microdilution (Sensititre commercial plates) according to the National Committee for Clinical Laboratory Standards (NCCLS) methods (*26*). *S. pneumoniae* ATCC 49619 and strain R6 were used for quality control. Fluoroquinolone efflux was determined (*27*).

### Genetic Transformation

*S. pneumoniae* strain R6 was grown in a casein hydrolysate-based medium (AGCH) with 0.2% sucrose and used as recipient in transformation experiments (*20*). Cultures containing 4 x 10^6^ CFU/mL were treated with DNA at 0.15 µg/mL for 40 min at 30°C, then at 37°C for 90 min before plating on media plates containing 2.5 µg/mL of ciprofloxacin. Colonies were counted after 24 h growth at 37°C in a 5% CO_2_ atmosphere in AGCH medium with 1% agar.

### Southern Blot Analysis

Probes for *parC* and *parE* were amplified by polymerase chain reaction (PCR) of the R6 strain with oligonucleotides parCUP and parCDOWN, parEUP and parEDOWN, respectively (*25*). The *ant* probe was obtained by amplifying strain 3870 DNA (*22*) with oligonucleotides antUP and antDOWN (*25*). All probes were labeled with the Phototope-Star Detection Kit (New England Biolabs, Beverly, MA). Southern blot and hybridization followed the manufacturer's instructions.

### Pulsed-field Gel Electrophoresis (PFGE)

Genomic DNA embedded in agarose plugs was restricted with *Sma*I, and fragments were separated by PFGE in a CHEF-DRIII apparatus (Bio-Rad, Hercules, CA) (*28*). PFGE patterns were compared to 14 representative international pneumococcal clones of the Pneumococcal Molecular Epidemiology Network (*28*). Strains with patterns varying by three or fewer bands were considered to represent the same PFGE type (*29*).

### PCR Amplifications and DNA Sequence Determination

Oligonucleotides gyrA44 and gyrA170 (*13*) were used to amplify and sequence the *gyrA* QRDRs from chromosomal DNA. Amplifications were performed with 0.5 U of *Thermus thermophilus* thermostable DNA polymerase (Biotools, Madrid, Spain), 0.1 µg of chromosomal DNA, 1 µmol/L (each) of the synthetic oligonucleotide primers, and 0.2 mmol/L of each deoxynucleoside triphosphate (dNTP) in the buffer recommended by the manufacturers. Amplification was achieved with an initial cycle of 1 min denaturation at 94°C; 25 cycles of 30 s at 94°C, 45 s at 55°C, and 90 s polymerase extension step at 72°C; and a final 3-min 72°C extension step, followed by slow cooling to 4°C. Fragments including ParE residues 398–647, the intergenic *parE*–*parC* region, and ParC residues 1–152 were amplified from genomic DNA with oligonucleotides parE398 (*13*) and parC152 (*16*) in PCR reactions performed as previously described, except that 30 cycles of amplification with an extension step of 3 min were applied. All strains, except three, CipR-73, CipR-74, and CipR-75, which yielded fragments of 2.4, 3.0, and 2.9 kb, respectively, yielded 1.6-kb fragments. Those PCR fragments that contained both *parE* and *parC* QRDRs were purified by using MicroSpin S400 HR columns (Amersham Pharmacia Biotech, Pistcatway, NJ) and sequenced on both strands with oligonucleotides parE398, parE483 (*13*), parC50, and parC152 (*16*) with an Applied Biosystems Prism 377 DNA sequencer, according to protocols provided by the manufacturer.

### Statistical Analysis

The Fisher exact test (χ^2^ test) was used when appropriate. Comparisons were considered significant at p < 0.05.

## Results

Among 2,882 strains, 75 (2.6%) had ciprofloxacin MIC > 4 µg/mL by microdilution and were considered ciprofloxacin-resistant. Ten strains with initial ciprofloxacin MIC 4 µg/mL by agar-dilution showed MIC 1–2 µg/mL by microdilution. Among the 75 ciprofloxacin-resistant strains, 67% were isolated from sputum, whereas 19% were from blood, 13% from lower respiratory tract samples, and 1.3% from pus. Although ciprofloxacin-resistant strains belonged to 17 different serotypes, 6 serotypes accounted for 55 (73.3%) of the strains: 14 (14 strains), 23F (11 strains), 19F (11 strains), 6B (8 strains), 3 (6 strains), and 15A (5 strains). These serotypes were also the most frequent among the 2,807 ciprofloxacin-susceptible strains. No ciprofloxacin-resistance was found among isolates from pediatric patients <15 years, whereas the prevalence of resistance was 75 (3.9%) of 1,904 isolates from adult patients (>15 years), and 53 (7.2%) of 738 isolates from patients >65 years (p < 0.001). The frequencies of ciprofloxacin resistance were higher among noninvasive pneumococci than among invasive strains (61 [4.3%] of 1,429 vs. 14 [1.0%] of 1,453, p < 0.001).

An association between penicillin and ciprofloxacin resistance was observed, with ciprofloxacin-resistant strains distributed as follows: 17 (1.0%) of 1,658 penicillin-susceptible, 39 (4.1%) of 961 intermediate-resistant, and 19 (7.2%) of 263 resistant isolates (p < 0.001). Ciprofloxacin-resistant pneumococci were also more represented among macrolide-resistant isolates: 22 (1.2%) ciprofloxacin-resistant strains among 1,833 erythromycin-susceptible and 53 (5.1%) ciprofloxacin-resistant strains among 1,049 erythromycin-resistant (p < 0.001). Antimicrobial-drug resistance among the 2,807 ciprofloxacin-susceptible pneumococci was lower than those of ciprofloxacin-resistant strains (penicillin 41.4% vs. 73.3%, erythromycin 35.5% vs. 70.7%, tetracycline 33.6% vs. 69.3%, and chloramphenicol 15.4% vs. 44.0%, p < 0.001). Fifty-five (73.3%) of ciprofloxacin-resistant strains showed multidrug resistance (resistant to more than three chemically unrelated drugs), among them, 26 strains were resistant to six antimicrobial agents (penicillin, tetracycline, chloramphenicol, erythromycin, clindamycin, and co-trimoxazole).

Fourteen (18.7%) ciprofloxacin-resistant (MIC 4–8 µg/mL) strains were classified as low-level ciprofloxacin-resistant, and 61 (81.3%) strains were classified as high-level ciprofloxacin-resistant (MIC 16–128 µg/mL). The comparative in vitro activity of five fluoroquinolones (MIC_90_) against the ciprofloxacin-resistant strains was the following: gemifloxacin (0.5 µg/mL) > moxifloxacin (4 µg/mL) > gatifloxacin (8 µg/mL) > levofloxacin (32 µg/mL) > ciprofloxacin (64 µg/mL) (Table 1). Gemifloxacin was the most active fluoroquinolone tested by using the NCCLS breakpoint (*26*), 49.3% of ciprofloxacin-resistant strains were nonsusceptible to this antimicrobial drug. Among the 14 low-level ciprofloxacin-resistant isolates, 12 were susceptible to levofloxacin, 13 to gatifloxacin, and all were susceptible to moxifloxacin and gemifloxacin according to NCCLS criteria (*26*). However, all but one of these low-level ciprofloxacin-resistant strains had first-step *parC* mutations that would favor the appearance of high-level resistant strains. Among the 61 high-level ciprofloxacin-resistant strains, all showed cross-resistance to levofloxacin and gatifloxacin and all but one to moxifloxacin. Twenty four (39.3%) of 61 high-level ciprofloxacin-resistant strains had gemifloxacin MIC 0.12 µg/mL, which could be considered susceptible according to NCCLS (*26*), although they showed double mutations (*parC* or *parE* and *gyrA*). One of 75 ciprofloxacin-resistant strains, strain CipR-71, with MIC 8 µg/mL, had an efflux mechanism as the single cause of resistance (see below).

**Table 1:** In vitro activity of 13 antimicrobial drugs against 75 ciprofloxacin-resistant *Streptococcus pneumoniae* isolates^a^

Drug	MIC_50_ (µg/mL)	MIC_90_ (µg/mL)	MIC range (µg/mL)	Susceptible breakpoints	%S	%I	%R	%I+R
Penicillin	1	2	0.03–8	<0.06	26.7	48.0	25.3	73.3
Amoxicillin	1	4	0.06–16	<2	80.0	12.0	8.0	20.0^b^
Cefotaxime	0.5	1	0.03–8	<1	92.1	5.3	2.6	7.9^b^
				<0.5	56.1	36.0	7.9	43.9^c^
Erythromycin	128	128	0.06–>256	<0.25	29.3	0.0	70.7	70.7
Clindamycin	128	128	0.06–>256	<0.25	37.3	0.0	62.7	62.7
Tetracycline	32	64	0.12–64	<2	30.7	2.7	66.6	69.3
Chloramphenicol	2	16	2–32	<4	56.0		44.0	44.0
Cotrimoxazole	>4/76	>4/76	0.5/9.5–>4/76	<0.5/9.5	32.0	4.0	64.0	68.0
Ciprofloxacin^d^	32	64	4–128	NA	NA	NA	NA	
Levofloxacin	16	32	1–64	<2	16.0	2.7	81.3	84.0
Gatifloxacin	4	8	0.5–16	<1	17.3	2.7	80.0	82.7
Moxifloxacin	2	4	0.12–8	<1	20.0	37.3	42.7	80.0
Gemifloxacin	0.12	0.5	0.06–2	<0.12	50.7	28.0	21.3	49.3

^a^S, susceptible; I, intermediate; and R, resistant, according to National Committee for Clinical Laboratory Standards (NCCLS) 2004 interpretative criteria; NA, not available. ^b^NCCLS 2004 nonmeningitis criteria. ^c^NCCLS 2004 meningitis criteria. ^d^Ciprofloxacin resistance defined as >4 µg/mL by Chen et al. (4).

The *parC*, *parE*, and *gyrA* QRDRs of 85 strains (75 ciprofloxacin-resistant strains and 10 strains with ciprofloxacin MIC 1–2 µg/mL) were characterized. Most resistant strains (70 of 75) showed low nucleotide sequence variations (<1%), but five strains had high variations (>4%) in at least one of their QRDRs (Figure). These strains would have a mosaic structure in those genes showing such a QRDR sequence variation (*25*): two strains in *parC*, one in *parC* + *parE*, and two in *parC* + *parE* + *gyrA*. Mosaic *parC* genes had the N91D change, and mosaic *gyrA* genes had the S114G GyrA change (Figure) that is also present in other ciprofloxacin-resistant *S. pneumoniae* strains with mosaic genes and in both ciprofloxacin-susceptible and -resistant VGS (*25*), which indicates their recombinational origin. As expected (*25*), strains with mosaic *parE* + *parC* genes had also an *ant*-like gene in the intergenic *parE*–*parC* region (Figure).

**Figure:**
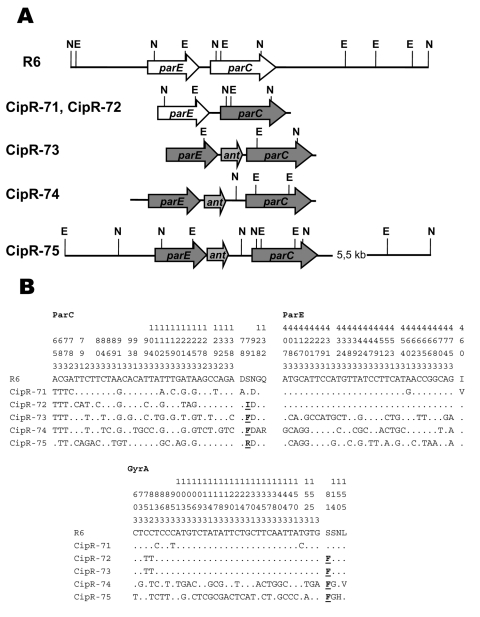
Genetic organization of the *parE*–*parC* region of *Streptococcus pneumoniae* mosaic strains and nucleotide sequence variations in the ParC, ParE, and GyrA quinolone resistance-determining regions. A) Structure as deduced from Southern blot experiments and nucleotide sequence analyses. E, *Eco*RV; N, *Nco*I. The *parE* and *parC* genes with a mosaic structure are shown with darker gray arrows. B) Nucleotides present at each polymorphic site are shown for strain R6, but only sites that differ are shown for the other strains. Amino acid changes involved in fluoroquinolone resistance are shown in **boldface** and underlined. Codon numbers are indicated in vertical format above the sequences. Positions 1, 2, and 3 in the fourth row refer to the first, second, and third nucleotides in the codon.

All low-level ciprofloxacin-resistant strains had mutations producing amino acid changes in ParC, whereas 92% of high-level resistant strains had double (ParC + GyrA or ParE + GyrA) and 8% had triple mutations (two changes in ParC + GyrA, ParC + ParE + GyrA, ParC + two changes in GyrA). Classical mutations known to be involved in fluoroquinolone resistance were found in all but two resistant strains with mosaic *parC* genes (Table 2): D78A (strain CipR-71) and S79R (strain CipR-75). Genetic transformation experiments showed that the S79R change was involved in ciprofloxacin resistance and that the D78A change was not. CipR-71 and CipR-75 chromosomal DNA transformed the susceptible R6 strain to resistance with high efficiency (>1 x 10^4^ transformants per microgram of DNA). The analysis of two transformants of each experiment showed that, while transformants obtained with CipR-71 DNA (ParC D78A) did not carry *parC* mutations, those obtained with CipR-75 (ParC S79R) carried the same nucleotide changes in *parC* as the donor strain.

**Table 2:** Fluoroquinolone MIC of 85 strains and amino acid changes in their DNA topoisomerase genes^a^

No. strains	Amino acid substitution			MIC (µg/mL)				
	ParC	Par E	GyrA	CIP	LVX	GAT	MXF	GEMI
10	None	None	None	1–2	1–2	0.12–0.5	0.12–0.25	0.015–0.03
1	None	None	None	8	2	0.5	0.12	0.06
8	S79F	None	None	4–8	2–4	0.5–2	0.12–0.5	0.03–0.06
3	S79Y	None	None	4–8	2–4	0.5	0.25	0.03–0.06
1	D83G	None	None	4	1	0.5	0.25	0.03
1	D83Y	None	None	4	1	0.5	0.25	0.03
1	S79A	None	S81F	16	8	4	2	0.12
21	S79F	None	S81F	32–128	8–32	4–8	2–4	0.12–1
9	S79F	None	S81Y	32–64	8–16	4–8	1–4	0.12–0.25
6	S79F	None	E85K	32–64	16–64	4–16	2–8	0.25–2
6	S79Y	None	S81F	32–64	8–64	4–8	2–4	0.12–0.25
1	S79Y	None	S81Y	32	16	8	4	0.12
2	D83N	None	S81F	32–64	8–16	2–4	2	0.12–0.25
1	D83N	None	S81F	32	16	8	4	0.5
3	None	D435N	S81F	16–32	8–16	4–8	2–4	0.12–0.25
1	None	D435N	S81Y	16	16	4	2	0.12
1	S79Y, D83N	None	E85K	64	32	8	4	1
3	S79F	D435N	E85K	64	64	8–16	8	0.5
1	S79Y	D435N	E85K	64	32	8	4	0.5
1	S79F	None	S81F, E85A	64	64	16	8	1
1	S79I	None	S81F	32	16	4	2	0.12
1	S79F	None	S81F	32	16	4	4	0.5
1	S79F	None	S81F	32	8	4	2	0.25
1	S79R	None	S81F	32	16	8	4	0.5

^a^Only changes involved in resistance are shown, and underlining indicates that the residue is located in a gene with a mosaic structure. Additional amino acid changes, not involved in resistance, were: ParC D78A (the CipR-71 strain with no mutations that has a mosaic *parC* gene), R36C (3 strains), ParC K137N (27 strains), ParC N91D (the three strains with mosaic *parC* genes), ParE I460V (40 strains), ParE453S (1 strain), GyrA V88I (3 strains), GyrA S114G (the two strains with mosaic *gyrA* genes), and GyrA N150H (one of the two strains with a mosaic *gyrA* gene). CIP, ciprofloxacin; LVX, levofloxacin; GAT, gatifloxacin; MXF, moxifloxacin; GEMI, gemifloxacin.

When PCR products carrying ParE residues 398–647, the intergenic *parE*–*parC* region, and ParC residues 1–152 (including ParE and ParC QRDRs) were used as DNA donors, only those obtained from strain CipR-75 were able to transform R6 to ciprofloxacin resistance. The two transformants selected carried the ParC S79R change, and one of them also had the N91D change. These results showed that the S79R change is involved in the resistance phenotype of strain CipR-75 and that the D78A change of strain CipR-71 is not involved in resistance. Determining MIC to ciprofloxacin, norfloxacin, ethidium bromide, and acriflavine in the presence or absence of reserpine showed greater than fourfold MIC decreases in the presence of reserpine, both in the CipR-71 strain and in the two transformants obtained when chromosomal DNA was used as donor, which indicates the existence of an efflux mechanism (data not shown).

Although 36 different PFGE patterns were observed in the 75 ciprofloxacin-resistant strains, 48 strains can be grouped into nine PFGE patterns (Table 3): 11 strains belonged to Spain^23F^-1 clone, 8 strains to Spain^14^-5 clone, 6 strains to Spain^9V^-3 clone, 5 strains to Spain^6B^-2 clone, 5 strains to Sweden^15A^-25 clone, 4 strains to clone C of serotype 19F, 4 strains to clone D of serotype 19F, 3 strains to clone A of serotype 3 related to multilocus sequence type (MLST) 260 (*30*), and 2 strains to clone B of serotype 3 related to MLST 180 (*30*). Among 14 blood isolates, 12 different PFGE types were observed. Only three of these strains (CipR-1, -2, and -15) shared an identical PFGE (clone A of serotype 3), although they had different *parE* polymorphisms and different resistance patterns (Table 2).

**Table 3:** Summary of phenotypic characteristics and changes in the QRDR among the most prevalent pulsed-field gel electrophoresis patterns of ciprofloxacin-resistant strains^a^

PFGE	Strain	Serotype	Resistance pattern	aa and nt changes in QRDR		
				ParC	ParE	GyrA
Referent	R6	NT	S	None	None	None
Spain^23F^-1	CipR-8	23F	PEClTCSxTCp	G128 (GGT), K137N, **S79F**	I460V, I476 (ATT)	Y75 (TAT)
	CipR-5	19A^b^	PTCSxTCp	G128 (GGT), K137N, **S79F**	I460V, I476 (ATT)	Y75 (TAT)
	CipR-9	23F	PEClTCSxTCp	G128 (GGT), K137N, **S79Y**	I460V, I476 (ATT)	Y75 (TAT)
	CipR-12	23F	PTCCp	G128 (GGT), K137N, **D83G**	I460V, I476 (ATT)	Y75 (TAT)
	CipR-30, -31, -32, -33	23F	PEClTCSxTCp	G128 (GGT), K137N, **S79F**	I460V, I476 (ATT)	Y75 (TAT), **S81F**
	CipR-48, -49	23F	PEClTCSxTCp	G128 (GGT), K137N, **S79F**	I460V, I476 (ATT)	Y75 (TAT), **E85K**
	CipR-73	19A^b^	EClTSxTCp	**S79F**	None	Y75 (TAT), **S81F**
Spain^14^-5	CipR-24	14	PEClTCSxTCp	**S79F**	*None*	Y75 (TAT), **S81F**
	CipR-41	14	PEClTCSxTCp	**S79F**	None	Y75 (TAT), **S81Y**
	CipR-55	14	PEClTCSxTCp	**S79Y**	None	Y75 (TAT), **S81F**
	CipR-66, -67	14	PEClTCSxTCp	**S79F**	**D435N**	Y75 (TAT), **E85K**
	CipR-38, -39, -40	14	PEClTCSxTCp	**S79F**	None	Y75 (TAT), V88I, **S81Y**
Spain^9V^-3	CipR-10	9V	PSxTCp	K137N, **S79Y**	I460V	Y75 (TAT)
	CipR-14	14^b^	PESxTCp	K137N, **S79A**	I460V	Y75 (TAT), **S81F**
	CipR-51	14^b^	PSxTCp	K137N, **S79Y**	I460V	Y75 (TAT), **S81F**
	CipR-20	9V	PSxTCp	K137N, **S79F**	I460V	Y75 (TAT), **S81F**
	CipR-28	19F^b^	PSxTCp	K137N, **S79F**	I460V	Y75 (TAT), **S81F**
	CipR-58	14^b^	PSxTCp	K137N, **D83N**	I460V	Y75 (TAT), **S81F**
Spain^6B^-2	CipR-3	6B	PEClTSxTCp	**S79F**	None	Y75 (TAT)
	CipR-17, -18, -19	6B	PEClTCSxTCp	**S79F**	None	Y75 (TAT), **S81F**
	CipR-72	6B	PEClTSxTCp	**S79F**	None	Y75 (TAT), **S81F**
Sweden^15A^-25	CipR-4	15A	PEClTCp	G77 (GGA), **S79F**	I460V	Y75 (TAT)
	CipR-64	15A	PEClTCp	G77 (GGA)	I460V, **D435N**	Y75 (TAT), **S81Y**
	CipR-60	15A	PEClTCp	G77 (GGA), **D83Y**	I460V	Y75 (TAT), **E85K**
	CipR-45, -50	15A	PEClTCp	G77 (GGA), **S79F**	I460V	Y75 (TAT), **E85K**
C	CipR-59	19F	PTCSxTCp	**D83N**	None	Y75 (TAT), **S81F**
	CipR-43, -44	19F	PTCSxTCp	**S79F**	None	Y75 (TAT), **S81Y**
	CipR-65	19F	PEClTCSxTCp	**S79Y**, **D83N**	None	Y75 (TAT), **E85K**
D	CipR-57	19F	PEClTCp	G77 (GGA), **S79Y**	I460V, P454S	Y75 (TAT), **S81Y**
	CipR-29	19F	PEClTCCp	G77 (GGA), **S79F**	I460V	Y75 (TAT), **S81F**
	CipR-46, -47	19F	PEClTCp	G77 (GGA), **S79F**	I460V	Y75 (TAT), **E85K**
A	CipR-1	3	Cp	R95C, **S79F**	I460V	Y75 (TAT), H104 (CAC)
	CipR-2	3	Cp	R95C, **S79F**	None	Y75 (TAT), H104 (CAC)
	CipR-15	3	TCCp	R95C, **S79F**	None	Y75 (TAT), H104 (CAC), **S81F**
B	CipR-61	3	Cp	None	I460V, **D435N**	Y75 (TAT), **S81F**
	CipR-52	3	ECp	**S79Y**	I460V	Y75 (TAT), **S81F**

^a^QRDR, quinolone-resistance determining regions; PFGE, pulse-field gel electrophoresis *Sma*I patterns; aa, amino acid; nt, nucleotide; S, susceptible to all antibiotics tested; P, resistant to penicillin (MIC 0.12–4 µg/mL); T, resistant to tetracycline (MIC >4 µg/mL); C, resistant to chloramphenicol (MIC >8 µg/mL); E, resistant to erythromycin (MIC >0.5 µg/mL); Cl, resistant to clindamycin (MIC >0.5 µg/mL); SxT, resistant to trimethropin-sulfamethoxazole (MIC >4/76 µg/mL); Cp, resistant to ciprofloxacin (MIC >4 µg/mL). Residue changes involved in fluoroquinolone resistance are showed in **boldface**, and underlining indicates that the residue is located in a gene with a mosaic structure. A, PFGE type related to serotype 3 with MLST 260; B, PFGE type related to serotype 3 with MLST 180; NT, nontypeable. ^b^Capsular switching.

One of the strains with a mosaic *parC* gene belonged to the Spain^6B^-2 clone, and one strain with mosaic *parC* and *parE* genes belonged to the Spain^23F^-1 clone. Capsular switching was observed in three strains of serotype 14 with the Spain^9V^-3 clone, one strain of serotype 19F with Spain^9V^-3 clone, and two strains of serotype 19A with Spain^23F^-1 clone. In general, strains that shared the same PFGE pattern shared identical polymorphisms on their DNA topoisomerase QRDRs with respect to the sequence of the R6 strain (Table 3). For instance, all strains of the Spain^9V^-3 clone had identical polymorphisms, the same found in the ATCC 700671 strain representative of this clone (*14*). All but one of the strains belonging to the Spain^23F^-1 clone had two polymorphisms in ParC (K137N and a change in codon G128: GGT instead of GGC), two polymorphisms in ParE (I460V and a change in codon I476: ATT instead of ATC), and a change in codon Y75 of GyrA (TAT instead of TAC). Identical polymorphisms were found in the ATCC 700669 strain representative of the Spain^23F^-1 clone (data not shown); the only exception was the Spain^23F^-1 strain CipR-73 that had *parC* and *parE* mosaic genes (Table 3). Other exceptions were three strains of the Spain^14^-5 clone (CipR-38, CipR-39, and CipR-40) that had an additional GyrA polymorphism (V88I), which were isolated from three patients temporally related in the same hospital, and two other strains, CipR-1 of clone A of serotype 3 and CipR-57 of clone D of serotype 19F that showed different polymorphisms in ParE (Table 3).

## Discussion

The worldwide prevalence of fluoroquinolone resistance in *S. pneumoniae* is low, although it varies over time, geographic region, age group, and origin of isolates (*1**,**4**,**6**,**31*). The overall incidence of ciprofloxacin-resistant *S. pneumoniae* strains in this study was 2.6%, lower than that reported in previous studies (3%–7%) (*4**,**5*) conducted in Spain in which noninvasive isolates were predominant. In agreement with those previous studies, noninvasive isolates in this study displayed ciprofloxacin resistance as high as 4.3%. A higher prevalence (7.2%) was seen in patients >65 years, which also agreed with previous reports (*4**,**6*), and which possibly reflects increased fluoroquinolone use in this group of patients. The prevalence of ciprofloxacin resistance seen in this study (2.6%) is similar to the 2.5% found among unselected isolates of Bellvitge Hospital (Barcelona) in 2002 (J. Liñares, pers. comm.), but it is lower than the 6.3% found in Donostia Hospital (San Sebastian) (E. Pérez-Trallero, pers. comm.).

A significant relation between resistance to penicillin or macrolides and resistance to ciprofloxacin was observed (Table 1), in agreement with previous reports (*4**,**6*). Penicillin- and multidrug-resistant pneumococci have been common in Spain since the 1980s; these strains primarily belong to serogroup 19 and to four multiresistant epidemic clones: Spain^23F^-1, Spain^6B^-2, Spain^9V^-3, and Spain^14^-5 (*30**,**32**,**33*). The emergence of fluoroquinolone resistance among strains of these clones has been described (*33**–**36*), and in our study, resistance occurred mainly among those four widely disseminated clones (Table 3). Since neither temporal nor geographic relationships were found among most patients infected by these four clones, this finding could reflect the high prevalence of these clones in the Spanish population, as suggested previously (*36*), rather than the spreading of resistant clones. Exceptions were five strains of the Spain^14^-5 clone that could be grouped in two temporal clusters. One cluster accounted for two strains (CipR-66 and -67) collected in the same laboratory, with identical polymorphisms and triple mutations in QRDRs. The second cluster accounted for three identical strains of the Spain^14^-5 clone (CipR-38, -39 and -40), which were recovered from three patients at a laboratory from San Sebastian in a 29-day period. These strains had an additional GyrA polymorphism (V88I) not present in other Spain^14^-5 clone strains and may represent a variant of this clone (*33*). Nevertheless, the epidemiologic features of these international clones (*2**,**37**,**38*) suggest that dissemination of ciprofloxacin resistance through these isolates is a plausible scenario and may predict a rapid increase of resistance in *S. pneumoniae* in countries with an increasing use of fluoroquinolones. Also plausible is that recombinant ciprofloxacin-resistant isolates with mosaic DNA topoisomerase genes belonging to the Spain^23F^-1 and Spain^6B^-2 international clones (Table 3) have disseminated and that these isolates have acquired resistance mutations by horizontal transfer from VGS. Although a low prevalence (5 [6.7%] of 75) of *S. pneumoniae* strains with mosaic *parC* genes among the ciprofloxacin-resistant strains was observed, the existence of these kind of strains (Figure) is an indication that recombination has occurred between resistant VGS and *S. pneumoniae*, including the prevalent pneumococcal clones. In addition, five resistant strains of the Sweden^15A^-25 clone, another multiresistant clone previously reported as susceptible (*28*), were found (Table 3).

Although newer fluoroquinolones have enhanced in vitro activity against *S. pneumoniae* and are less likely to select for resistant isolates, their use to treat respiratory tract infections merits special attention. More than 20 reports of levofloxacin treatment failure have been concurrent with the development of resistance during or after therapy. In some cases, strains susceptible to levofloxacin but with a first-step *parC* mutation, as a consequence of previous quinolone use, were present before therapy was initiated. Identifying these strains will help avoid therapeutic failures. Since fluoroquinolone resistance is more frequent in the elderly with chronic respiratory diseases who have had long-term quinolone therapy (*4**,**6**,**10**–**12**,**14*), recent use of these drugs should contraindicate further fluoroquinolone treatment.

The use of NCCLS susceptibility breakpoints for newer fluoroquinolones underestimates a high proportion of low-level ciprofloxacin-resistant strains with first-step mutations whose detection should be improved by decreasing those breakpoints as previously suggested (*11**,**39*). In the present study, which followed NCCLS criteria (*26*), 12 (85%) of 14 low-level ciprofloxacin-resistant strains with a single *parC* mutation were susceptible to levofloxacin (MIC 1–2 µg/mL). These data agree with those of other authors who found *parC* mutations in 59% of the strains with levofloxacin MIC 2 µg/mL (*39*). Using a ciprofloxacin resistance breakpoint MIC >4 µg/mL, as was used by Chen et al. (*6*), would improve detection of these mutant strains. In fact, in our study, no strains with first-step mutations and ciprofloxacin MIC 2 µg/mL were detected, although other authors have found these mutations in up to 29% of this type of strain (*39*). Although gemifloxacin was the most active quinolone studied, and 39.3% of high-level ciprofloxacin resistance with double mutations were gemifloxacin-susceptible with the NCCLS breakpoint, a patient infected by such strains should not be treated with any quinolone. To avoid treatment failures, using microbiologic breakpoints for quinolone susceptibility would be more prudent than using clinical breakpoints. If fluoroquinolones are widely and indiscriminately used, resistance to fluoroquinolones in *S. pneumoniae* may become a problem in the near future.
